# Time of Progression to Osteopenia/Osteoporosis in Chronically HIV-Infected Patients: Screening DXA Scan

**DOI:** 10.1371/journal.pone.0046031

**Published:** 2012-10-08

**Authors:** Eugenia Negredo, Anna Bonjoch, Moisés Gómez-Mateu, Carla Estany, Jordi Puig, Nuria Perez-Alvarez, Joaquin Rosales, Silvana di Gregorio, Luis del Rio, Guadalupe Gómez, Bonaventura Clotet

**Affiliations:** 1 Lluita contra la SIDA Foundation, Hospital Universitari Germans Trias i Pujol, Universitat Autònoma de Barcelona, Barcelona, Spain; 2 Statistics and Operations Research Department, Universitat Politècnica de Catalunya, Barcelona, Spain; 3 CETIR Centre Mèdic, Barcelona, Spain; 4 Irsicaixa Foundation, Barcelona, Spain; University of Southern California, United States of America

## Abstract

**Background:**

Algorithms for bone mineral density (BMD) management in HIV-infected patients are lacking. Our objective was to assess how often a dual-energy x-ray absorptiometry (DXA) scan should be performed by assessing time of progression to osteopenia/osteoporosis.

**Methods:**

All DXA scans performed between 2000 and 2009 from HIV-infected patients with at least two DXA were included. Time to an event (osteopenia and osteoporosis) was assessed using the Kaplan–Meier method. Strata (tertiles) were defined using baseline minimum T scores. Differences between strata in time to an event were compared with the log-rank test.

**Results:**

Of 391 patients (1,639 DXAs), 49.6% had osteopenia and 21.7% osteoporosis at their first DXA scan. Of the 112 (28.6%) with normal BMD, 35.7% progressed to osteopenia; median progression time was 6.7 years. These patients were stratified: “low-risk" (baseline minimum T score >−0.2 SD), “middle-risk" (between −0.2 and −0.6 SD), and “high-risk" (from −0.6 to −1 SD); median progression time to osteopenia was 8.7, >7.2, and 1.7 years, respectively (*p*<0.0001). Of patients with osteopenia, 23.7% progressed to osteoporosis; median progression time was >8.5 years. Progression time was >8.2 years in “low-risk" tertile (T score between −1.1 and −1.6 SD), >8.5 years in “middle-risk" (between −1.6 and −2), and 3.2 years in “high-risk" (from −2 to −2.4) (*p<0.0001*).

**Conclusions:**

Our results may help to define the BMD testing interval. The lowest T score tertiles would suggest recommending a subsequent DXA in 1–2 years; in the highest tertiles, ≥6 years. Early intervention in patients with bone demineralization could reduce fracture–related morbidity/mortality.

## Introduction

Bone strength is the result of bone mineral density (BMD) and bone microarchitecture. A decrease in BMD leads to deterioration of microarchitecture, and is determined by the intensity of bone remodeling. These changes lead to critical damage and porosity that weaken bone and increase the probability of fractures.[1]

Many factors lead to accelerated bone loss, and HIV infection is one of the most recently identified [2], [3], [4]. The results of several studies confirm a higher incidence of osteopenia and osteoporosis in HIV-infected patients than in HIV-negative individuals. In fact, up to 25% of infected patients fulfill the criteria for osteoporosis and up to 50% fulfill those of osteopenia [2], [3], [4], [5]. Accelerated bone demineralization in this population is due to an additional number of factors such as the HIV infection itself and the chronic inflammatory status, by increasing apoptosis of osteoblast cells and promotion of osteoclastic activity [6], [7], [8], [9], [10], [11], [12], [13], [14], [15], the exposure to specific antiretroviral agents [2], [13], [16], [17], [18] and the immune reconstitution [14], [19], [20], together with traditional risk factors, such as vitamin D insufficiency, a secondary cause of osteoporosis that is very prevalent among HIV-infected patients. [21]

In addition, the first available longitudinal studies revealed that HIV-infected patients experience more rapid bone loss [5], [19], [20] and more frequent fractures than HIV-negative individuals. [20], [22], [23], [24]

The high associated morbidity and mortality of bone fractures [25] means that optimal monitoring of bone mineralization should be offered to patients at high risk of low BMD, such as the HIV-infected population.

Dual-energy X-ray absorptiometry (DXA) is the method of choice for the assessment of BMD. Most clinicians attending HIV-infected patients agree that this group should undergo DXA screening, especially those patients with traditional risk factors for bone loss. [26], [27] However, the optimal BMD testing intervals remains undetermined.

This study aims to determine how often a DXA scan should be performed in chronically HIV-infected patients by assessing the time of progression to osteopenia or osteoporosis in a large cohort.

## Methods

### Study design, population, and objective

We performed a retrospective longitudinal observational study of all DXA scans from HIV-infected patients who attended our HIV Unit between January 2000 and December 2009.

Of a total of 671 patients with at least one DXA scan, 391 had at least two DXA scans performed during the study period. These scans were included in the present analysis. The study was performed according to the stipulations of the Declaration of Helsinki, and all patients gave their written informed consent for their medical information to be used for purposes of scientific research in accordance with the ethical committee of the participating site. A more detailed description of the methodology applied has been described elsewhere. [2]

The objective of the study was to determine the time of progression to bone loss defined as time from normal BMD to osteopenia and from osteopenia to osteoporosis.

Osteopenia and osteoporosis were defined following the World Health Organization (WHO) criteria, as follows: normal, when the T score by DXA was higher than −1.0 SD, osteopenia when the T score was between −1.0 SD and −2.5 SD, and osteoporosis when the T score was less than −2.5 SD. [28]

### Assessments

A total of 1,639 DXA scans from 391 patients were included in the present analysis.

The mean BMD and T score (comparison with normal reference values for young adults expressed as standard deviation units) for the lumbar spine and femoral neck were measured using the same DXA device (Lunar Prodigy, GE Healthcare, Belgium) at an external center (CETIR Grup Mèdic, Barcelona, Spain).

Epidemiological and HIV-related data (time since diagnosis of HIV infection, time on antiretroviral treatment, viral load, CD4 T-cell count, nadir CD4 T-cell count, total time on treatment, total time on protease inhibitors, total time on tenofovir, baseline antiretroviral treatment, time with viral suppression, and hepatitis B or C coinfection) were collected from the database of our HIV Unit at time of the first DXA scan.

### Statistical analysis

A descriptive analysis of the main quantitative and categorical variables was performed.

We calculated time of progression in weeks from normal BMD to osteopenia. The calculation was based on those individuals whose BMD was considered normal according to a first DXA scan and who fulfilled the criteria for osteopenia in a subsequent scan. The same procedure was followed to compute time from osteopenia to osteoporosis (ie, patients with osteopenia in a first DXA scan who progressed to osteoporosis).

Patients with normal BMD at baseline and at least 3 DXA scans who progressed from normal BMD status to osteopenia and later to osteoporosis were considered for both time from normal BMD status to osteopenia and time from osteopenia to osteoporosis.

The time to an event, namely, change of category (normal to osteopenia, osteopenia to osteoporosis), was known only for those patients who presented the event before the last DXA scan. For the remaining patients, the only known data were that the time to the event was greater than the observation time. This approach is known as administrative censoring, and the incomplete data are termed right-censored. [29]

Additionally, data were stratified by tertiles of the baseline T score. These distinct groups represent the BMD baseline status. The initial value is related to the time to an event, assuming that the T score is a monotone and decreases over time.

Survival was analyzed using the Kaplan-Meier estimator; the log-rank test was used to compare strata. If the last time of observation is not censored, then the curve fall to zero and an exact value of time is provided while, if the last observed time is censored, the survival remains constant from the last time value and result is provided as “>than" this value.

All analyses were performed using the R software, and all reported *p* values are two-tailed.

## Results

Epidemiological and clinical data are summarized in Table 1.

**Table 1:** Epidemiological and clinical data at first DXA scan.

**Gender**, male, n (%)	284 (73%)
**Menopause**, n (%)	10 (9.5%)
**Age,** years	39.4 (34.9; 44.2)
**BMI**, kg/m^2^	22.5 (20.9; 24.6)
**Calcium intake**, g/d	16 (4%)
**Hepatitis B/C coinfection**, n (%)	93 (29%)
**Concomitant therapy**, n (%)	
** Hormone treatment**, n (%)	3 (1%)
** Alendronate**, n (%)	7 (2%)
**Time since HIV diagnosis**, years	7.9 (3.7; 12.1)
**Current CD4+**, **absolute value,** cells/µL	514 (348.5; 746.5)
**Nadir CD4+**, cells/µL	219.5 (106.8; 318.3)
**Suppressed viral load**, n (%)	268 (68.5%)
**Proportion of time with suppressed viral load**, n (%)	30.6 (11.7; 49.3)
**Naïve**, n (%)	48 (12%)
**Time on antiretroviral therapy**, years	4.8 (1.5; 8.5)
**Current use of protease inhibitors**, n (%)	171 (50%)
**Time on protease inhibitors**, years	2.4 (1.2; 4.1)
**Current use of tenofovir**, n (%)	173 (51%)
**Time on tenofovir**, years	1 (0.3; 2.3)
**Creatinine**, µmol/L	87 (76; 97.5)

Values are expressed as median (IQR) or number (%).

At the first DXA scan, 112 patients of 391 (28.6%) had normal BMD, 194 (49.6%) fulfilled the criteria for osteopenia, and 85 (21.7%) fulfilled the criteria for osteoporosis.

The mean (SD) number of DXA scans per patient (DXA scans/patient) was 4 (2); 100% of patients had 2 DXA scans, 67% had 3 DXA scans, 48% had 4 DXA scans, and 35% had 5 or more. No differences were observed in mean (SD) DXA scans/patient after stratification taking the first DXA as normal (5 (3) DXA/patient), osteopenia (4 (3) DXA/patient), and osteoporosis (4 (2) DXA/patient) (p = 0.111).

Median (IQR) time between DXA scans was 33.71 weeks (25.1; 56) (0.65 years); median (IQR) time between the first and the last DXA scans was 133.1 weeks (62.7; 276.4) (2.6 years). No differences were detected in median (IQR) time between the first and the last DXA scans after stratification according to the first DXA scan (data not shown).

### Change from normal BMD at baseline to osteopenia

Median follow-up for the 112 patients from this group was 152 weeks (59.1; 258) (2.9 years). Forty of these 112 patients (35.7%) progressed to osteopenia/osteoporosis.

**Figure 1:**
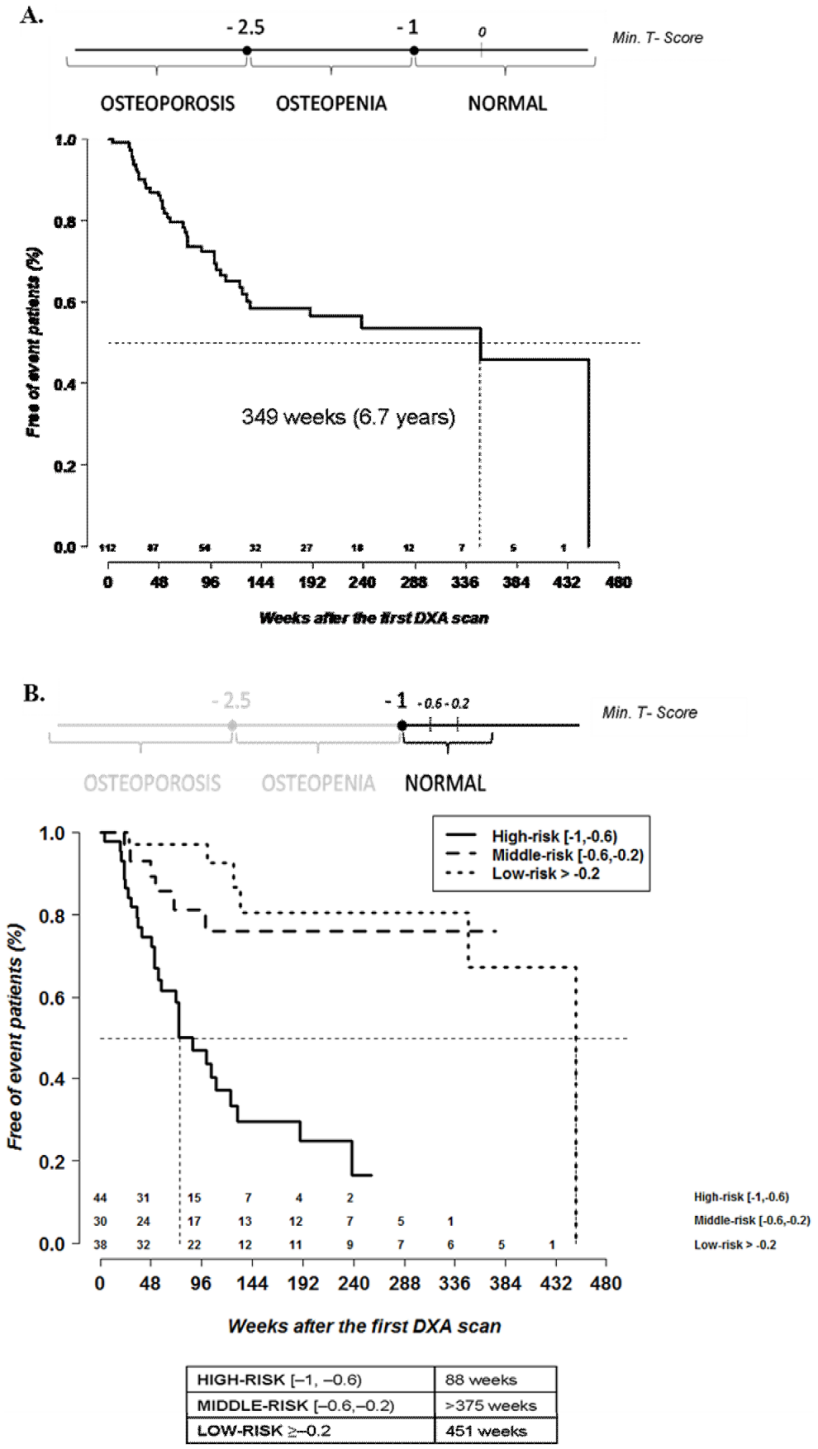
Time of progression. Time of progression from normal bone mineral density to osteopenia overall (n = 112 patients) (A) and after stratification according to minimum baseline T score (“low-risk", “middle-risk", and “high-risk") (B).

The survival analysis showed a median (IQR) time of progression to osteopenia of 349 weeks (74; 451) (6.7 years) (Figure 1.A).

Patients with normal BMD at baseline were stratified in tertiles according to the minimum T score of their first DXA, as follows: “low-risk" tertile when the minimum T score was >−0.2 SD (38 patients, 33.9%), “middle-risk" tertile when the minimum T score was between −0.2 and −0.6 SD (30 patients, 26.8%) and “high-risk" tertile when the minimum T score was between −0.6 and −1 SD (44 patients, 39.3%) (Figure 1.B).

The three groups were similar with respect to age (p = 0.95), gender (p = 0.72), time with infection (p = 0.064), percentage of patients with viral suppression (p = 0.81), and CD4 T-cell count (p = 0.359).

The survival analysis revealed significant differences in time of progression after stratification by tertiles. Median (IQR) time of progression overall was 451 weeks (349; 451) (8.7 years) in patients from the “low-risk" tertile, >375 weeks (>375; >375) (>7.2 years) in those who were in the “middle-risk" tertile, and 88 weeks (39; 190) (1.7 years) in those who were in the “high-risk" tertile (*p*<0.0001) (Figure 1.B).

Table 2 shows those of the 112 patients whose status progressed from normal to osteopenia/osteoporosis after stratification by baseline minimum T score (“low-risk", “middle-risk", and “high-risk").

**Table 2:** Patients (n = 112) who progressed from normal bone mineral density to osteopenia/osteoporosis.

*Follow-up (weeks)*	HIGH-RISK	MIDDLE-RISK	LOW-RISK
48	25%	11%	3%
96	53%	19%	3%
144	70%	24%	20%
192	75%	24%	20%
240	84%	24%	20%
288		24%	20%
336		24%	20%
384			33%
432			33%
480			100%

**Figure 2:**
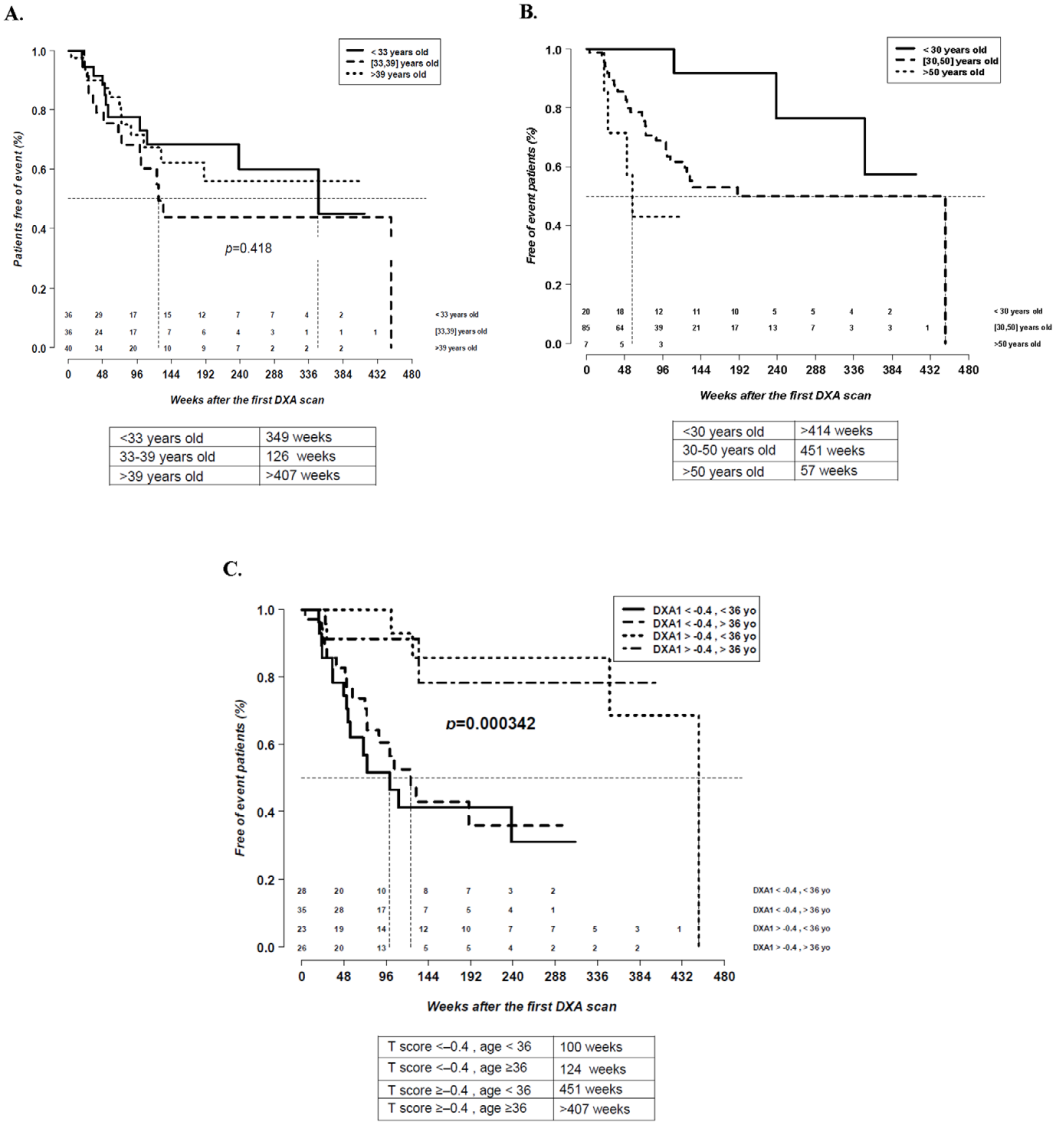
Survival analysis by age. Survival analysis in patients with normal bone mineral density at baseline stratified by age (<33 years, 33–38 years and >38 years [A] and <30 years, 30–50 years, and >50 years [B]) and by both age and T score (less or higher than −0.4 and less or higher than 36 years [C]).

When patients were stratified by age tertiles in the same group, differences in time of progression were observed when we applied the following tertiles: those aged <30 years (20 patients, 17.9%), those aged 30–50 years (85 patients, 75.9%), and those aged >50 years (7 patients, 6.3%) (*p* = 0.016), but not when we applied the tertiles <33, 33–39, and >39 years (p = 0.418) (Figure 2.A and 2.B).

When patients were stratified by both the minimum T score of baseline DXA scan and by age, the median time of progression was significantly shorter in tertiles with a T score <−0.4, independently of age, than in those with a T score ≥−0.4 (*p* = 0.0003) (Figure 2.C).

No statistically significant differences were observed when patients were classified according to gender: time of progression was >392 weeks (130; >392) (>7.5 years) in women (n = 35) and 238 weeks (72; 451) (4.6 years) in men (n = 77) (*p* = 0.136).

### Change from osteopenia at baseline to osteoporosis

A total of 211 patients had osteopenia at baseline; of these, 194 presented osteopenia at their first DXA scan and the remaining 17 had a normal DXA result at baseline, progressed to osteopenia in a subsequent DXA, and underwent additional DXA scans.

Median follow-up for the 211 patients from this group was 128 weeks (60.4; 279) (2.7 years). Fifty of these 211 patients (23.7%) progressed to osteoporosis.

**Figure 3:**
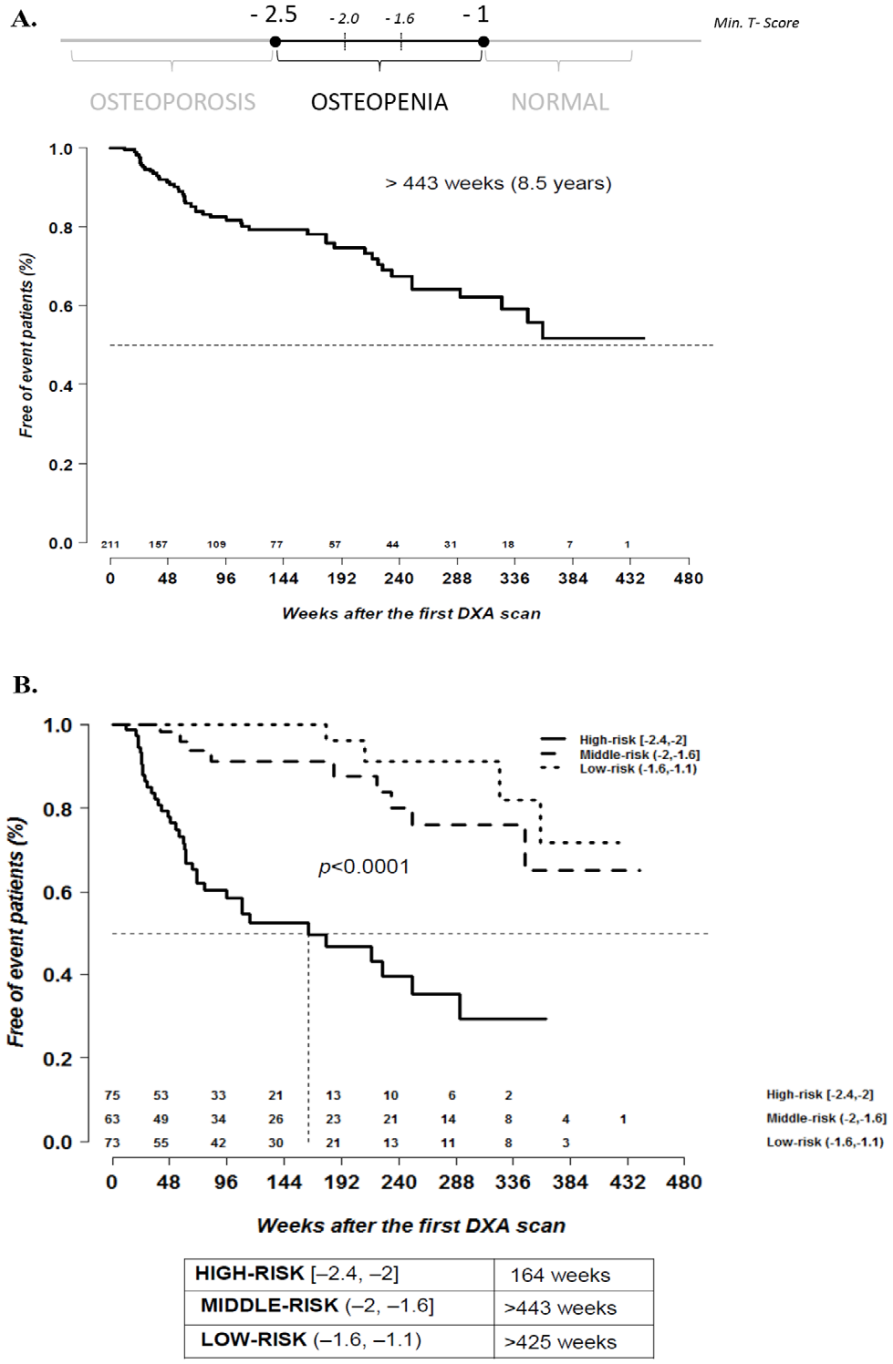
Time of progression to osteoporosis. Time of progression from osteopenia to osteoporosis overall (n = 211 patients) (A) and after stratification by baseline minimum T score (“low-risk", “middle-risk", and “high-risk") (B).

Survival analysis showed a median (IQR) time of progression to osteoporosis of >443 weeks (186; >443) (>8.5 years) (Figure 3.A).

When the 211 patients were stratified according to the minimum T score of the first DXA scan, 73 (34.6%) were classed as “low-risk" (between −1.6 and −1.1 SD), 63 (29.9%) as “middle-risk" (between −2 and −1.6 SD and 75 (35.5%) as “high-risk" (between −2.4 and −2 SD) (Figure 3).

The 3 groups were similar with respect to age (*p* = 0.106), gender (*p* = 0.103), percentage of patients with viral suppression (p = 0.602), and CD4 T-cell count (*p* = 0.739), but not by time with infection, which was higher in patients from the “high-risk" tertile with respect to the “low-risk" tertile (9.4 years vs 7.8 years, *p* = 0.043).

The survival analysis showed significant differences in time of progression to osteoporosis between the 3 groups, as follows: median (IQR) time of progression of >425 weeks (359; >425), (>8.2 years) in the “low-risk" tertile, >443 weeks (347; >443) (>8.5 years) in the “middle-risk" tertile, and 164 weeks (53; 364) (3.2 years) in the “high-risk" tertile (*p*<0.0001) (Figure 3.B)

Table 3 shows those of the 211 patients who progressed from osteopenia to osteoporosis stratified by minimum T score (“low-risk", “middle-risk", and “high-risk").

**Table 3:** Patients (n = 211) who progressed from osteopenia to osteoporosis (B) after stratification according to minimum T score (“low-risk", “middle-risk" and “high-risk").

*Follow-up (weeks)*	HIGH-RISK	MIDDLE-RISK	LOW-RISK
48	22%	2%	0%
96	40%	9%	0%
144	60%	9%	0%
192	71%	13%	4%
240	71%	20%	9%
288	71%	24%	9%
336	71%	24%	18%
384		36%	28%
432		36%	
480			

**Figure 4:**
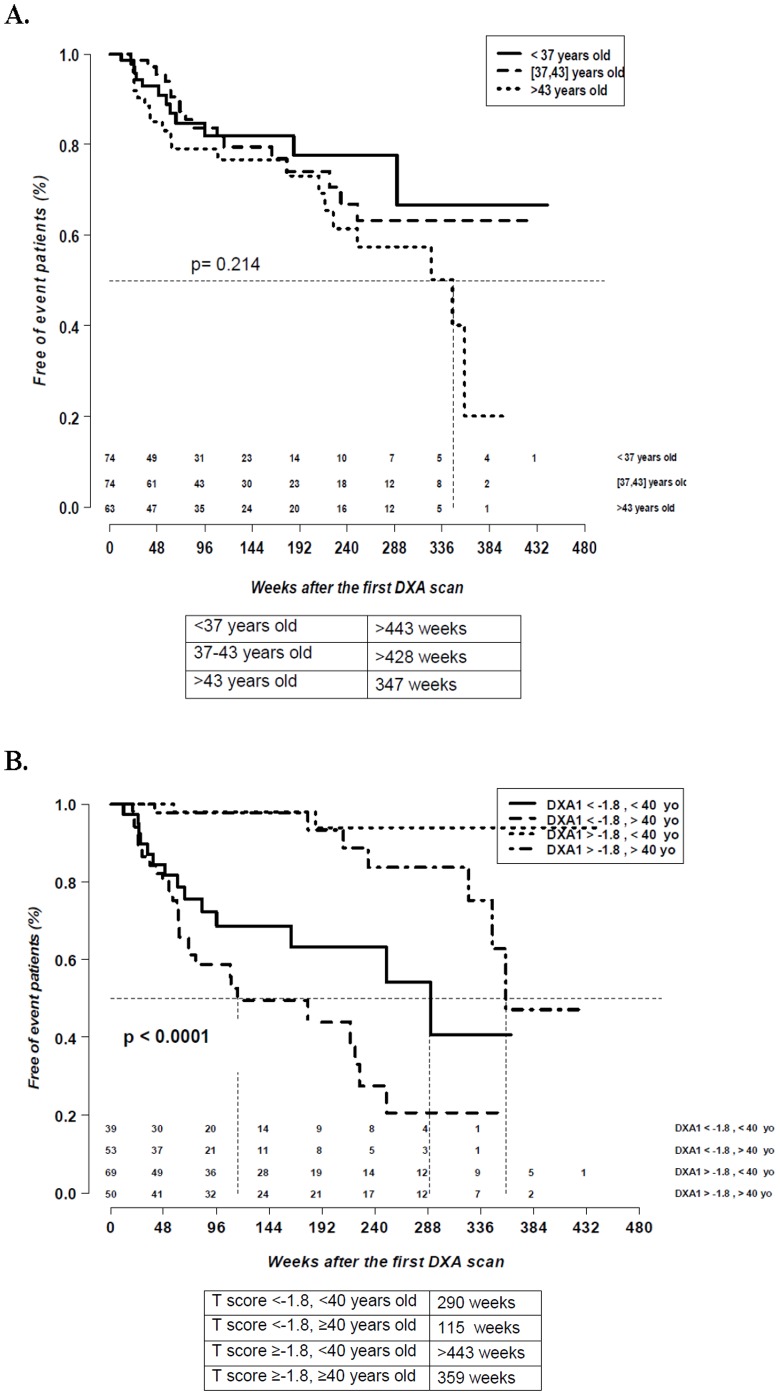
Time of progression stratified by age and T score. Time of progression stratified by age: <37 years, 37–43 years and >43 years (A) and by both the minimum values of the T score and age (B).

When patients were stratified by age tertiles, no significant differences were seen for <37 years (74 patients, 35%), 37–43 years (74 patients, 35%), or >43 years of age (63 patients, 30%) (*p* = 0.214) (Figure 4.A).

When patients were stratified by both the minimum values of the T score of the DXA scan and age, median time of progression was significantly shorter in the group with a T score <−1.8 and age >40 years (*p*<0.0001) (Figure 4.B).

No differences were seen when patients were classified according to gender: median time of progression (IQR) was 359 (234; >443) weeks (6.9 years) in women (n = 60) and >428 weeks (179; >428) (>8.2 years) in men (n = 151) (*p* = 0.465).

As for patients with osteoporosis in the first DXA scan, 24.7% improved (osteoporosis to osteopenia or normal BMD), while 75.3% remained unchanged.

## Discussion

Among our chronically HIV-infected patients, nearly one-third of those whose first DXA scan showed normal BMD progressed to osteopenia. Median time of progression to osteopenia was almost 7 years, but in those patients with normal BMD but whose baseline minimum T score was in the “high-risk" tertile, this progression was much faster (<2 years). Similarly, osteopenia progressed to osteoporosis in a quarter of patients. Although time of progression was more than 8 years, for patients with a minimum baseline T score <−2 SD (lower tertile) it was 3 years.

Alterations in bone strength predispose to fragility fractures, which are associated with increased morbidity and mortality in the HIV-negative population. [1], [24], [25], [30]

Prevention of osteoporotic fractures, then, is of major interest from a public health perspective. In HIV-infected patients, osteoporosis is probably underdiagnosed. Adequate tools should be designed to identify patients at high risk for low BMD, who would be candidates for BMD screening by DXA. In the last few years, many efforts have been made to identify risk factors for bone loss—other than traditional factors—among HIV-infected patients. [2], [27], [31] Age (>50 years), the menopause, other secondary causes of low BMD, and a personal or family (parents) history of fragility fractures, together with exposure to some antiretroviral drugs (tenofovir and boosted protease inhibitors), are relevant risk factors that speak in favor of screening with DXA. [31] The optimal BMD testing intervals using DXA scans, however, has not been established.

Data have been published on the rate of progression to bone loss [32], [33], [34], although our results are the first to show the time of progression to osteopenia/osteoporosis, information that is essential for monitoring. We observed that baseline T score, divided into tertiles, enabled us to establish time of progression of bone loss more accurately than the simple classification according to the WHO criteria (normal, osteopenia, and osteoporosis). According to our results, time of progression to osteopenia in patients with normal BMD at the first DXA scan was enormously different depending on the tertile in which the patient was classified at baseline: from more than 8 years to less than 2 years according to whether the T score was in the low-risk tertile (>−0.2 SD) or if it was in the lowest tertile (from −0.6 to −1 SD), respectively. Similarly, the time of progression to osteoporosis varied widely among patients with osteopenia in the first DXA scan according to the tertile: 8 years for those whose minimum T score was in the higher tertile (between −1.1 and 1.6 SD), but only 3 years for those whose minimum T score was in the lowest tertile (from −2 to −2.4 SD). Ourt results are consistent with recent published data in older HIV-negative women. In this analysis of data from 4,957 women, baseline T score was the most determinant of a BMD testing interval; osteoporosis developed in 15 years for women with normal BMD or mild osteopenia (T score, greater than −1.5), 5 years for women with moderate osteopenia (T score from −1.5 to −1.99) and 1 year for women with advanced osteopenia (T score from −2.0 to −2.49) [35].

Current recommendations on the study of bone disease for HIV care providers mainly include information on candidates for BMD monitoring by DXA, [22], [29] but provide very little information on the optimal frequency of subsequent DXA scans, based on the WHO categories of BMD. [31] According to our data, a reasonable proposal would be to set the frequency of DXA according to the tertile: patients in the lowest T score tertiles (defined as a T score from −0.6 to −1 in patients with normal BMD according to the WHO criteria, and from −2 to −2.4 among those with osteopenia) should undergo another DXA scan more frequently than those in the other 2 tertiles. A subsequent DXA scan should be recommended as soon as 1–2 years later for those in the lower tertiles, even when baseline BMD had been classified as normal; this frequency will help to diagnose early changes in BMD status (from normal BMD to osteopenia or from osteopenia to osteoporosis) and, in the case of progression, enable appropriate measures to be taken. Patients in the other 2 tertiles (T score from −0.6 to >−0.2 in patients with normal BMD and from −1.1 to −2 among those with osteopenia) could be assessed by a DXA scan after at least 6–7 years, even in those whose baseline BMD fulfilled the criteria for osteopenia. Since it is unlikely that a patient would progress in less than 7–8 years, more frequent DXA scanning would not affect management, although it would increase costs and inconvenience to patients.

Given our results, similar recommendations on the frequency of DXA scan should be implemented according to age, providing that the decision is based on the T score. Although age affected bone loss, when patients were classified according to their initial T score (tertiles), BMD status gave more information on the progression of bone loss. In clinical terms, however, the negative effect of age on risk of osteoporotic fracture, independently of BMD, has been reported, suggesting that both age and BMD should be taken into consideration [36] The lack of elderly patients prevents us from assessing this issue in greater depth.

As for gender, although no statistically significant differences were detected between men and women, variations in the time of progression to osteopenia/osteoporosis could be clinically relevant: those men with a normal baseline BMD, included in the high-risk tertile, progressed more rapidly to osteopenia than women (median of progression of 4.6 years in men and >7.5 years in women). This finding could suggest the need for special care and more rigorous monitoring of men from this group. These data are consistent with other published results showing the higher prevalence of low BMD and fragility fractures in men than in premenopausal women. [25]

As a retrospective observational study, our analysis presents some limitations. Variables were recorded using clinical documents as the source. Consequently, the first DXA scan for each patient did not correspond to a defined baseline; however, since at that time DXA scan was only performed when patients were seen for first time in our unit or in the context of clinical assays (metabolic studies, new antiretroviral strategies, antiretroviral-naive patients…), subjects included in the analysis seem to be a good representation of our population. Additionally, the time from normal BMD to osteopenia is only known to lie within the time interval between the first DXA with a value <−1 SD and the time immediately before this scan; the time from osteopenia to osteoporosis is only known to lie within the time interval between the first DXA with a value <−2.5 SD and the time immediately before this scan. Our analysis imputed the right end of this interval, that is the time with the first DXA <−1 SD as the time to osteopenia and <−2.5 SD as the time to osteoporosis. A statistical analysis taking into account the random nature of the intervals, where the change of status occurs, could diminish the bias due to this finding. [37] However, it is our belief that the effect of these limitations on the conclusions would be negligible. Finally, the retrospective nature of the analysis avoids knowing the presence of some clinical factors that may contribute to a different bone loss progression or bone recovery (steroid use, menopause, hypogonadism, bisphosphonate use, vitamin D or calcium supplementation, etc).

To conclude, in HIV-infected patients, the use of screening thresholds based on the minimum T scores (tertiles) provided by DXA is more efficient for predicting time of progression to osteopenia/osteoporosis and deciding on BMD testing intervals than the WHO criteria. The lowest minimum T-score tertiles indicate the recommendation of a subsequent DXA scan in 1–2 years, while in the highest tertiles, this interval could be 6 years or more. Since management algorithms are lacking, ours and other data are necessary to facilitate the design of suitable guidelines for this population. Early intervention in patients with bone demineralization could reduce the high morbidity and mortality associated with osteoporotic fractures.
